# A biologically oriented algorithm for spatial sound segregation

**DOI:** 10.3389/fnins.2022.1004071

**Published:** 2022-10-14

**Authors:** Kenny F. Chou, Alexander D. Boyd, Virginia Best, H. Steven Colburn, Kamal Sen

**Affiliations:** ^1^Department of Biomedical Engineering, Boston University, Boston, MA, United States; ^2^Department of Speech, Language and Hearing Sciences, Boston University, Boston, MA, United States

**Keywords:** multitalker speech perception, sound (audio) processing, sound segregation, cocktail party problem, binaural hearing, spatial listening, hearing loss

## Abstract

Listening in an acoustically cluttered scene remains a difficult task for both machines and hearing-impaired listeners. Normal-hearing listeners accomplish this task with relative ease by segregating the scene into its constituent sound sources, then selecting and attending to a target source. An assistive listening device that mimics the biological mechanisms underlying this behavior may provide an effective solution for those with difficulty listening in acoustically cluttered environments (e.g., a cocktail party). Here, we present a binaural sound segregation algorithm based on a hierarchical network model of the auditory system. In the algorithm, binaural sound inputs first drive populations of neurons tuned to specific spatial locations and frequencies. The spiking response of neurons in the output layer are then reconstructed into audible waveforms *via* a novel reconstruction method. We evaluate the performance of the algorithm with a speech-on-speech intelligibility task in normal-hearing listeners. This two-microphone-input algorithm is shown to provide listeners with perceptual benefit similar to that of a 16-microphone acoustic beamformer. These results demonstrate the promise of this biologically inspired algorithm for enhancing selective listening in challenging multi-talker scenes.

## Introduction

Attending to a single conversation partner in the presence of multiple distracting talkers (i.e., the Cocktail Party Problem, CPP) is a complicated and difficult task for machines and humans (Haykin and Chen, 2005; McDermott, 2009; Qian et al., 2018). While some listeners can accomplish this task with relative ease, other groups of listeners report great difficulty—such as those with sensorineural hearing loss (Kochkin, 2000, 2007; Shinn-Cunningham and Best, 2008), cochlear implant users (Bernstein et al., 2016; Goupell et al., 2016, 2018; Litovsky et al., 2017), subgroups of children (Dhamani et al., 2013), persons with aphasia (Villard and Kidd, 2019) and adults with “hidden hearing loss” (Pichora-Fuller et al., 2017; Shinn-Cunningham, 2017; Parthasarathy et al., 2019). At a cocktail party, talkers are distributed in space. Listeners use spatial cues (i.e., interaural timing and level differences, or ITDs and ILDs, respectively) for sound localization. Additionally, normal-hearing listeners appear to make use of spatial cues in addition to a variety of other talker-related cues, to perceptually segregate the competing talkers and attend to the one of most interest. Indeed, spatial listening has been shown to provide enormous benefit to listeners in cocktail-party scenarios (Litovsky, 2012; Rennies and Kidd, 2018).

Sound processing algorithms can be designed with the distinct goals of sound localization or spatial sound segregation. Specifically, spatial processing plays a key role in several sound segregation algorithms that aim to help hearing-impaired listeners overcome the CPP. For example, acoustic beamforming techniques utilize multiple microphones to selectively enhance signals from a desired direction (Gannot et al., 2017; Chiariotti et al., 2019), and are often employed in hearing aids (Greenberg and Zurek, 2001; Chung, 2004; Doclo et al., 2010; Picou et al., 2014; Launer et al., 2016). Machine learning approaches such as clustering using Gaussian mixture models (MESSL) (Mandel et al., 2010) and deep neural networks (DNN) (Wang et al., 2014), among others, also make use of ITDs and ILDs to localize the target sound.

The ability of human listeners with normal hearing to solve the CPP is quite remarkable. Many animals, too, appear to have robust solutions to their own versions of the CPP (Bee and Micheyl, 2008). Unlike beamformers, which benefit from using microphone arrays, humans and animals require only two inputs—the left and right ear. These listeners are also able to solve the CPP in novel and unpredictable settings, a challenge for algorithms that rely on supervised learning (Bentsen et al., 2018; Wang and Chen, 2018). This raises the idea that spatially selective algorithms may benefit from incorporating insights from the human and/or animal brain. From a practical standpoint, biological processing, which is based on neural spikes, also has practical advantages that make it uniquely suited for always-on, portable devices such as hearing aids. Spike-based processing is computationally efficient and can be implemented with higher temporal resolution than algorithms operating on sampled waveforms (Ghosh-Dastidar and Adeli, 2009), especially when implemented on specialized neuromorphic hardware (Roy et al., 2019).

We recently proposed a biologically inspired algorithm for sound processing. The primary goal of this algorithm was to use spatial cues to perform sound segregation and selection, not sound localization. In this algorithm, sound mixtures were segregated by spatially selective model neurons, and selection was achieved by selective integration *via* a cortical network model (Chou et al., 2019). For the tested conditions, which included a frontal target talker and two symmetrically placed masker talkers, the algorithm showed segregation performance similar to MESSL and DNN, and provided proof-of-concept for a biologically based speech processing algorithm. However, the algorithm operated in the spiking domain, and employed a linear decoding algorithm to recover the target speech (Mesgarani et al., 2009), which resulted in low objective speech intelligibility. Like many typical beamformers, the algorithm also did not preserve binaural cues in the output, which can be particularly problematic in multitalker mixtures (Best et al., 2017; Wang et al., 2020). These drawbacks limited its practical use for applications in hearing-assistive devices and machine hearing.

In this study, we present a new biologically oriented sound segregation algorithm (BOSSA) that overcomes specific limitations of our previous algorithm. We introduce a time-frequency mask estimation method for decoding processed neural spikes that improves the quality of recovered target speech compared to the current standard approach (Mesgarani et al., 2009). We compared the proposed two-channel algorithm to a 16-microphone super-directional beamformer, using both objective measures and human psychophysics, and showed equivalent performance. Our algorithm overcomes some of the challenges faced by current state-of-the-art technologies, and provides an alternative, biologically based approach to the CPP.

## Algorithm design and implementation

**FIGURE 1:**
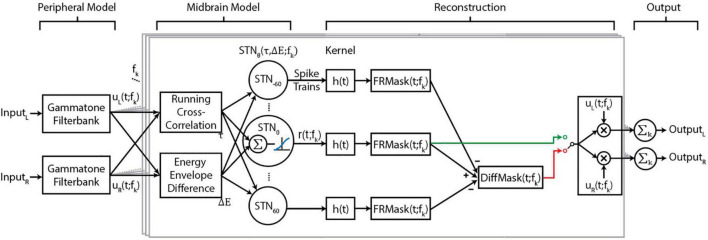
Flow diagram of the proposed algorithm. Central boxes, outlined in gray, show processing for a single-frequency band. The functions u_L_ (t; f_*k*_) and u_L_ (t; f_*k*_) are the narrowband signals of the left and right input channels for each frequency channel, and f_k_ denotes the k^th^ frequency channel. The midbrain model is based on spatially tuned neurons (STNs), where each STN has a “best” ITD and ILD, denoted **τ** and **ΔE**, respectively. The best ITD and ILD values of a neuron depend on the direction θ and frequency f_k_ to which the STN is tuned. h(t) represents the reconstruction kernel that converts spike trains to waveforms. We implemented two masks, FRMask (green line) and DiffMask (red line), either of which could be used for reconstruction, as indicated by the switch, The implementation of DiffMask in our analysis involves five sets of STNs, where θ ∈ {0, ± 30, ± 60}; however, other implementations of the model may involve different sets of θ.

The proposed BOSSA algorithm contains three modules (Figure 1) that together generate neural output patterns that are inputs to the target-reconstruction stage. The first module resembles peripheral filtering by the cochlea. The second module performs spatial segregation by constructing model neurons sensitive to specific spatial cues in narrow frequency bands. Ensembles of neurons then encode sounds that share the same spatial cues. In the third module, the spiking activity of output neurons are decoded into intelligible waveforms using a novel reconstruction approach. All modules are implemented in MATLAB (MathWorks, Natick, MA, United States).

### Peripheral filtering

Left and right channels of the input audio are filtered with a gammatone equivalent-rectangular-bandwidth (ERB) filterbank, implemented using the auditory toolbox in MATLAB (Slaney, 1998). The bandwidths were calculated using $ERB={\left[{\left(f_{c}/Q\right)}^{x}+b^{x}\right]}^{\frac{1}{x}}$ with parameters Q = 9.26449 (Glasberg and Moore, 1990), minimum bandwidth (b) = 24.7 Hz, order (x) = 1. The filterbank used here has 64 channels with center frequencies ranging from *f*_1_ = 200 *Hz* to *f*_64_ = 20 *kHz*. The filterbank outputs are two sets of 64 channels of narrowband signals, *u*_*L*_ (*t*; *f*_*k*_) and *u*_*R*_ (*t*; *f*_*k*_), corresponding to the left and right channels, respectively.

### Midbrain model

First, binaural cues of input signals are extracted based on a model of the barn-owl inferior colliculus (Fischer et al., 2009). ITD was calculated as a short-time running cross correlation between the energy-normalized *u*_*L*_ (*t*; *f*_*k*_) and *u*_*R*_ (*t*; *f*_*k*_) and ILD as the energy envelope difference between *u*_*L*_ (*t*; *f*_*k*_) and *u*_*R*_ (*t*; *f*_*k*_). Gain modulation steps matching those used in Fischer et al. (2009) were applied to the filterbank outputs such that the inputs to the cross correlation calculation, (*u*_*L*_ (*t*; *f*_*k*_) and *u*_*R*_ (*t*; *f*_*k*_)), varied as a linear function of stimulus level. Further gain control applied during the cross correlation calculation in conjunction with a logarithmic energy envelope calculation resulted in an approximately stimulus level invariant ILD representation. For a detailed description of the mathematical operations and their physiological basis, we refer interested readers to Fischer et al. (2009).

We then constructed sets of spatially tuned neurons (STNs), where each set consists of 64 neurons tuned to *f_k_* of the previous module. The 64 neurons in each set are sensitive to the same specific direction θ in the horizontal plane (*STN*_θ_, Figure 1), and each neuron has specific parameters τ (θ; *f*_*k*_) and Δ*E* (θ, *f*_*k*_), corresponding to the ITD and ILD for that specific θ. Each neuron’s preferred time-lag τ was calculated using the Woodworth formulation (Woodworth, 1938), with the approximation that ITDs are independent of frequency. Preliminary studies found that using frequency-dependent ITD values, calculated as described by Fischer et al. (2009) or the ones described by Aaronson and Hartmann (2014), provided no benefit in terms of objective measures of algorithm performance. On the other hand, Δ*E* is frequency-dependent, and was derived by calculating the ILD of a narrow band noise placed at various azimuths. Directionality of the narrow band noise was imparted by convolving with Head Related Transfer Functions (HRTFs) of the Knowles Electronic Manikin for Acoustic Research (KEMAR) (Burkhard and Sachs, 1975; Algazi et al., 2001).

The responses of model neurons were then calculated as follows. If the stimulus energy envelope difference was within a preset range of the neuron’s preferred Δ*E*, then that energy-envelope difference was weighted by the energy envelope of either *u*_*L*_ (*t*; *f*_*k*_) or *u*_*R*_ (*t*; *f*_*k*_). The ITD and ILD components were combined additively at the subthreshold level and then transformed *via* a sigmoidal input-output non-linearity (i.e., an activation function) to obtain an instantaneous firing rate. Finally, a Poisson spiking generator was used to generate spike trains for each neuron [*r*_θ_ (*t*; *f*_*k*_), Figure 1]. This sequence of operations is expected to produce a multiplicative spiking response to ITD and ILD in each model neuron as explained in Fischer et al. (2009). These steps, including the activation function, were kept identical for all frequency channels. Parameters for the input-output nonlinearity were modified from a step-function to a sigmoidal function to increase the dynamic range of the model neurons’ firing rates.

**FIGURE 2:**
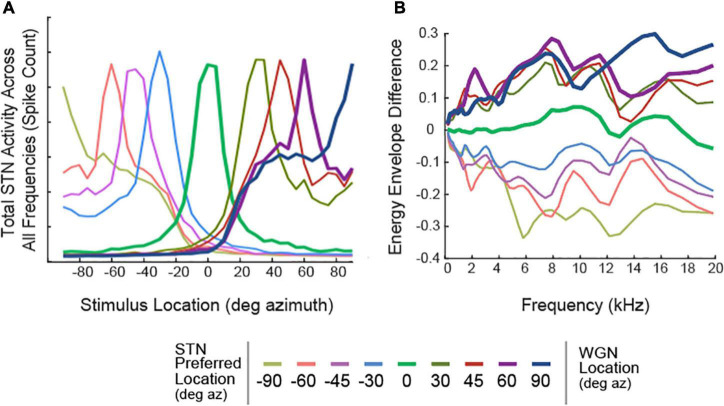
Spatial Tuning Characteristics of STNs. **(A)** Spatial Tuning Curves of STNs, represented as total neural activity in response to a White Gaussian Noise (WGN). Each colored line represents the total response from a set of STNs. Tuning curves of STNs tuned to 0°, 60°, and 90° azimuth are bolded. **(B)** Preferred ILDs of STNs, calculated using WGNs placed at various locations along the azimuthal plane.

The model can be implemented with any number and configuration of STNs. For illustrations of spatial tuning curves in Figure 2A, nine sets of STNs were constructed where θ ∈ {0°, ± 30°, ± 45°, ± 60°, ± 90°}. The ILDs used in generating the neuron spatial tuning curves are shown in Figure 2B, where each line represents Δ*E* (θ, *f*_*k*_) for a set of *STN*_θ_. All other results were obtained by constructing five sets of STNs, where θ ∈ {0°, ± 30°, ± 60°}.

### Stimulus reconstruction

The stimulus reconstruction module decodes ensembles of neural spikes into audible waveforms, using an approach similar to ideal time-frequency mask estimation (Wang, 2005). The concept of time-frequency masks can be summarized as follows: for a time-frequency representation of an audio mixture (e.g., spectrogram) consisting of a target and interferers, one can evaluate each element (i.e., time-frequency tile) of such a representation and determine whether the energy present is dominated by the target or the masker. If the target sound dominates, a value of unity (1) is assigned to that time-frequency tile, and zero (0) otherwise. This process creates an ideal binary mask. Alternatively, assigning the ratio of energies of the target to total energies in a time-frequency tile yields the ideal ratio mask (Srinivasan et al., 2006). One can then estimate the target sound by applying the mask to the sound mixture *via* element-wise multiplication. This process has been shown to recover the target with high fidelity in various types of noise (Wang, 2005). A key idea to both binary and ratio masks is the application of a gain factor to each time-frequency tile of a signal. In the proposed BOSSA algorithm we adopt a similar approach but calculate the gain factor for each time-frequency tile based solely on user-defined knowledge of the target location, as explained below.

The spiking responses from the spatially tuned neurons, *r* (*t*; *f*_*k*_), were convolved with a kernel, *h*(*t*), to calculate a smoothed, firing-rate-like measure. We set the kernel to be an alpha function: *h*(*t*) = *te*^−*t*/τ_*h*_^, a common function involved in modeling neural dynamics. We used a value of τ_*h*_ = 20 ms (see section Model Parameters) and the kernel was restricted to a length of 100 ms.

The same kernel was convolved with the spike trains of each frequency channel independently. The resulting firing rates of each set of STNs were treated as a non-binary time-frequency mask:


$$FRMask\;\left(t;f_{k}\right)=r\;\left(t;f_{k}\right)*h\left(t\right)$$

where * denotes convolution. We note that the FRMask is akin to a smoothed version of the firing rate. Thus, in theory, FRMask could be directly derived from the firing rate (without the need for spikes). However, the midbrain model can be used as a front-end to spiking network models, where the calculation of spikes is necessary (Chou et al., 2019). Thus, we kept this more versatile implementation.

The mask was then applied (i.e., point-multiplied) to the left and right channels of the original sound mixture. Then, we summed (without weighting) each frequency channel of the FRMask-filtered signal to obtain an audible, segregated waveform. We designated this result as *Ŝ*.


$${\hat{S}}_{j}=\sum_{k}FRMask\;\left(t;f_{k}\right)\cdot u_{j}\left(t;f_{k}\right),j\in \left\{L,R\right\}$$

This procedure resulted in a binaural signal and retained the natural spatial cues of the sound sources.

To reduce spatial leakage, we calculated a DiffMask by calculating FRMasks for each *STN*_θ_, then subtracting scaled versions of the off-center *STN*_θ_ from *STN*_0_, followed by rectification:


$$DiffMask=Max\;\left(FRMask_{0}-a\Sigma FRMask_{\theta },0\right)$$

where θ ∈ [± 30°, ± 60°] corresponds to the location of maskers in our experimental stimuli (see section “Psychophysical Experiment”). In this operation, each FRMask was first scaled to [0,1]. The scaling factor *a* was chosen to be 0.5 (see section “Model Parameters”) and was fixed across all frequencies and spatial channels to reduce the amount of computational complexity in the algorithm.

### Model parameters

Although a behavioral measure of algorithm performance using human psychophysics is the gold standard, such experiments are too time-consuming to explore model parameter variations. For practical reasons, most model parameters were fixed to biologically plausible values. We explored variations in the time-constant of the alpha function kernel (τ_*h*_), and the scaling factor for DiffMask (*a*). We chose the specific values of these parameters using an iterative process by trying a range of values, quantifying algorithm performance using the Short Time Objective Intelligibility (STOI) measure (Taal et al., 2010), and choosing parameters that produced the highest average STOI. STOI is an approximation of speech intelligibility, and ranges between 0 and 1. We do not claim that this approach produces an optimal set of parameters for reconstruction. However, objective measures combined with our behavioral results indicate that the parameter values we chose generated good reconstructions.

## Algorithm performance

### Spatial tuning characteristics

Spatial tuning responses of STNs were important predictors of the model’s segregation performance. We define “spatial tuning curves” as the spiking activity of STNs as a function of stimulus location. To construct spatial tuning curves, white Gaussian noise was convolved with anechoic KEMAR HRTFs, then presented to the algorithm. Figure 2 shows the responses of STNs combined across frequency channels. Ideally, STNs would only respond to stimuli from one specific direction. However, Figure 2 shows that all STNs also respond to off-target locations. For example, STNs tuned to 0° azimuth (Figure 2A, green curve) respond to stimuli at ±30° azimuth and even have a non-zero response to stimuli at ±90° azimuth. We refer to this property as “spatial leakage,” which occurs due to overlap in the bandpass filters as well as the fact that a given binaural cue can occur for stimuli from multiple locations (Figure 2B) and thus contain some ambiguity (Brainard et al., 1992).

### Spatial leakage

**FIGURE 3:**
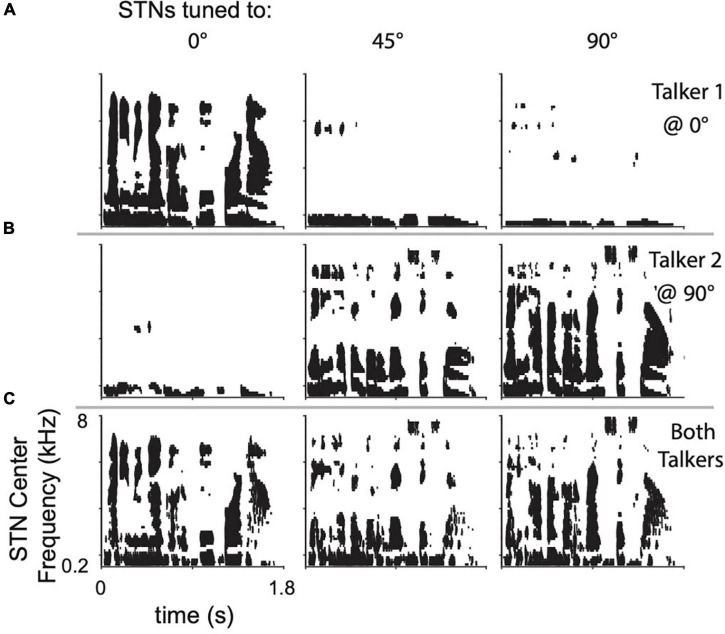
Raster plots of STN responses to **(A)** top row, a single sentence placed at 0° azimuth, **(B)** center row, a different sentence placed at 90° azimuth, and **(C)** bottom row, both sentences present at their respective locations. Columns show the STN responses when tuned to the location indicated.

Leakage across spatial channels limits the performance of the algorithm, especially when multiple sound sources are present. To demonstrate, two randomly selected sentences were presented individually to the model from 0° azimuth, 90° azimuth, or simultaneously from both locations. The responses of three set of STNs, tuned to 90°, 45°, and 0°, are shown as spike-rasters in Figure 3. Each row within a raster plot represents the spiking response from the neuron tuned to that particular frequency channel. Due to spatial leakage, all STNs respond to the single sentence placed at 0° or 90° (Figures 3A,B). When both sentences are present, ITDs and ILDs interact to produce complicated STN response patterns (Figure 3C). Spatial leakage limits the ability of STNs to respond to a single talker, since any one spatial channel contains information from other spatial channels. Lateral inhibition was designed to address the issue of spatial leakage by suppressing neural activation by off-target sound streams.

### DiffMask

**FIGURE 4:**
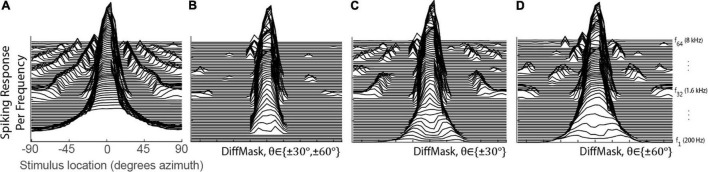
Spatial Tuning of the 0° STNs for before **(A)** and after **(B–D)** the DiffMask operation. Each line represents the spatial tuning curve of a single frequency-specific neuron within the set of STNs ranging from 200 to 8 kHz with ERB spacing. STN of the neuron tuned to the lowest frequency is placed on the bottom of the plots. STNs involved in the DiffMask operation are denoted in each subplot.

The DiffMask operation was inspired by lateral inhibition observed in biological networks. This operation was applied to the spatial tuning curves of 0° STNs to illustrate its sharpening effect on spatial tuning. Figure 4A shows the tuning curves prior to the DiffMask operation. Some neurons within the 0° STNs were activated by stimuli from as far away as 90° (see side peaks). After the DiffMask operation, spiking activity elicited by far-away stimuli was silenced, and side-peaks were suppressed considerably (Figure 4B). Using a subset of STNs during the DiffMask operation, such as those tuned to ±30° (Figure 4C) or ±60° (Figure 4D), did not suppress side-peaks as effectively as if both ±30° and ±60° were used.

## Psychophysical experiment

A psychophysical experiment was conducted to quantify the perceptual benefit provided by the algorithm for listeners with normal hearing. The performance of FRMask and DiffMask was compared against a 16-microphone super-directional beamformer, called BEAMAR (Kidd et al., 2015; Best et al., 2017). BEAMAR attenuates off-center sounds by combining the weighted output of 16 omni-directional microphones into a single channel, using an optimal-directivity algorithm (Stadler and Rabinowitz, 1993). BEAMAR does not process frequencies below 1 kHz in order to retain natural spatial cues in that frequency region. The combination of beamforming at high frequencies and natural binaural signals at low frequencies has been shown to provide a significant benefit to both normal-hearing and hearing-impaired listeners attending to a target speech sentence in a multi-talker mixture (Best et al., 2017).

### Participants

Participants in this study were eleven young normal-hearing listeners, ages 18–32. All listeners had symmetrical audiogram measurements between 0.25 and 8 kHz with hearing thresholds within 20 dB HL. Participants were paid for their participation and gave written informed consent. All procedures were approved by the Boston University Institutional Review Board (protocol 1301E).

### Stimuli

Five-word sentences were constructed from a corpus of monosyllabic words (Kidd et al., 2008), with the form [name-verb-number-adjective-noun] (e.g., “Sue found three red hats”). The corpus contains eight words in each of the five categories. Each word in each sentence was spoken by a different female talker, randomly chosen from a set of eight female talkers, without repetition. During each trial, a target sentence was mixed with four masker sentences, all constructed in the same manner. Words from the target and masker sentences were time-aligned, so that the words from each category shared the same onset. The design of these stimuli was intended to reduce the availability of voice and timing-related cues, and as such increase the listener’s use of spatial information to solve the task.

The five sentences were simulated to originate from five spatial locations: 0°, ±30°, and ±60° azimuth, by convolving each sentence with anechoic KEMAR HRTFs. The target sentence was always located at 0° azimuth. The four maskers were presented at 55 dB SPL from ±30°, and ±60° azimuth. The level of the target was varied to achieve target-to-masker ratios (TMRs) of –5, 0, and 5 dB.

Stimuli were processed using one of three methods: BEAMAR, FRMask, and DiffMask. A control condition was also included, in which stimuli were spatialized using KEMAR HRTFs to convey “natural” cues but were otherwise unprocessed.

### Procedures

Three blocks were presented for each of the four conditions, with each block containing five trials at each of the three TMRs (15 total trials per block). This resulted in 15 trials per TMR for each of the four processing conditions, and a total of 180 trials across all conditions. The order of presentation of TMRs within a block, and the order of blocks for each participant, were chosen at random. The experiment took approximately 1 h to complete.

Stimuli were controlled in MATLAB and presented *via* a real time processor and headphone driver (RP2.1 & HB7, Tucker Davis Technologies, Alachua, FL, United States) through a pair of headphones (Sennheiser HD265 Linear). The sound system was calibrated at the headphones with a sound meter (type 2250; Brüel & Kjær, Nærum, Denmark). Participants were seated in a double-walled sound-treated booth. A computer monitor inside the booth displayed a graphical user interface containing a grid of 40 words (five columns of eight words, each column corresponding to one position of the five word sentence). For each trial, participants were presented a sentence mixture and were instructed to listen for the target sentence located directly in front. They responded with a mouse by choosing one word from each column on the grid.

### Analysis

Each participant’s performance was evaluated by calculating the percentage of correctly answered keywords across all trials for a given condition. Psychometric functions were generated by plotting the percent correct as a function of TMR and fitting a logistic function to those data. Speech reception thresholds (SRTs), which are the TMRs corresponding to 50% correct, were extracted from each function using the psignifit toolbox (Schütt et al., 2016). Differences in SRTs between the natural condition and each of the processing conditions was taken to be the “benefit” provided by that processing method. Statistical analysis was done in Python using the statsmodels package (Seabold and Perktold, 2010).

## Results

**FIGURE 5:**
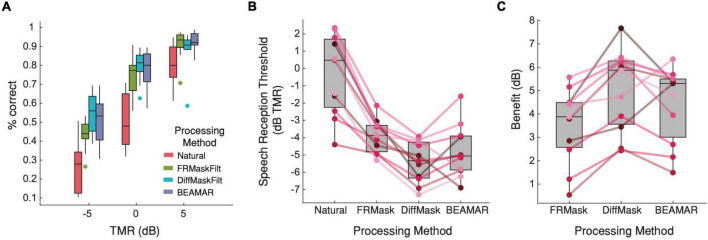
Behavioral evaluation results. **(A)** Average percent correct for each processing condition as a function of TMR. *n* = 11. Higher is better. **(B)** Average and individual subject speech reception threshold for each processing method. Lower is better. Solid lines connect the SRTs for each participant. **(C)** Average and individual subject perceptual benefits relative to the natural condition. Higher is better.

Figure 5A shows the percentage of correct responses for each TMR and processing method. A two-way repeated-measures ANOVA found a significant interaction between processing method and TMR on performance [*F*_(6,60)_ = 6.97, *p* < 0.001]. *Post hoc* pairwise comparisons using Tukey’s HSD test found significant differences between the natural condition and each of the three processing methods for all three TMRs (*p* < 0.001), suggesting that subjects significantly benefitted from listening to processed speech across all TMRs. At +5-dB TMR, performance was equivalent under all three processing conditions. However, at –5-dB and 0-dB TMR, performance was better for DiffMask than FRMask, and similar for DiffMask and BEAMAR. Figure 5B presents the same results in terms of SRTs, and Figure 5C shows the benefit (in dB) of each processing method relative to the natural condition. A one-way repeated measures ANOVA followed by Tukey’s multiple pairwise comparison showed that all three algorithms provided significant benefit to listeners (*p* < 0.001). Benefits provided by BEAMAR and DiffMask were not significantly different (*p* = 0.66). Out of the eleven listeners, two achieved the lowest SRT and gained the most benefit from BEAMAR, while nine achieved the lowest SRT and gained the most benefit from DiffMask.

## Discussion

Extensive research has been devoted to developing a solution for the CPP [for review, see Qian et al. (2018)], and many approaches benefit from using multiple microphones. For example, the performance of methods using independent component analysis degrades quickly as the number of sources exceeds the number of microphones (Hyvärinen et al., 2001). In acoustic beamforming, performance of the beamformer can be significantly improved by increasing the number of microphones used (Greenberg and Zurek, 2001; Greenberg et al., 2003). Although traditional beamformers produce a single-channel output, which cannot carry binaural information, a variety of spatial-cue preservation strategies have been proposed to overcome this limitation (Doclo et al., 2010; Best et al., 2017; Wang et al., 2020). Here we demonstrated that equivalent performance to a highly optimized beamformer (such as BEAMAR) may be possible using a biologically inspired algorithm that uses only two microphones placed in the ears. Our biologically oriented sound segregation (BOSSA) model provided a substantial benefit in a challenging cocktail party listening situation, and this benefit was larger than that provided by BEAMAR in the majority of our young, normal hearing participants. While this is a promising result, further work is needed to examine the benefits of BOSSA under a wider variety of scenarios and in other groups of listeners. Comparisons to other two-microphone solutions such as binaural beamformers (Doclo et al., 2010; Best et al., 2015), as well as deep-learning solutions that operate on two or even a single microphone (Roman et al., 2003; Healy et al., 2013), would also be interesting.

Spiking neural networks traditionally do not have applications in audio processing due to the lack of a method that produces intelligible, high-quality reconstructions. The “optimal prior” method of reconstruction is often used to obtain reconstructions from physiologically recoded neural responses (Bialek et al., 1991; Stanley et al., 1999; Mesgarani et al., 2009; Mesgarani and Chang, 2012), but produces single-audio-channel reconstructions of poor quality and intelligibility (Chou et al., 2019). The optimal prior method computes a linear filter between a training stimulus and the response of neuron ensembles, and filter needs to be re-trained if the underlying network changes. In contrast, the mask-based reconstruction method used in this study estimates time-frequency masks from spike trains. It is able to obtain reconstructions with much higher intelligibility and preserves spatial cues, all without the need for training. These properties enable rapid development of spiking neural network models for audio-related applications.

Within the biologically plausible algorithms we tested, the difference in performance between FRMask and DiffMask is noteworthy and interesting. The spatial tuning plots (Figure 2) quantify the tuning of a given spatial channel to a single sound as it is moved around the lateral spatial field which are reasonably well-tuned. Moreover, Figures 3A,B, for example, illustrate the response of the 0° channel to sounds presented at 0° and 90°. In this case, the 0° channel responded primarily to the frontal sound. By themselves, these plots do not suggest problems with spatial tuning and leakage. However, in our psychophysical experiments, we presented a target sound at 0° with four competing maskers from ±30° and ±60°, a far more challenging scenario. In such a scenario, spatial leakage is more significant, and refining/improving spatial tuning improves sound segregation, as demonstrated in the improvement with DiffMask over FRMask.

It is also worth noting that our algorithms were based on processing in the barn owl midbrain which contains a topographic map of space, whereas, in mammals, no such topographic map has been found. Despite this difference, the spatially tuned responses of neurons in the model could be leveraged to improve speech segregation performance in humans. This demonstrates that brain inspired algorithms based on non-human model systems can improve human perception and performance.

The work presented here represents a preliminary evaluation of the BOSSA model, and it identified a number of issues and limitations that deserve further investigation. While the formulation of DiffMask can sharpen the spatial tuning of the STNs, neurons tuned to frequencies below 300 Hz were completely silenced for the stimuli we tested (Figure 4B). Low spatial acuity in this frequency range results in a similar response at on and off target STNs. The off-target response scaling and summation that forms DiffMask then results in a complete subtraction of on-target activity below 300 Hz. Additionally, some side peaks still persist even after the DiffMask operation, implying that spatial leakage was not fully addressed. Different formulations of the DiffMask may address these shortcomings. Moreover, our DiffMask implementation used a specific number of off-target STNs at specific locations, which were aligned with the locations of makers in our experimental stimuli. Further works is needed to explore how DiffMask can be optimized to support arbitrary target and masker configurations, and how the resolution of the STNs affects model performance. We have avoided using deep-learning approaches in this study in favor of biological interpretability, but such approaches may help guide the optimization of DiffMask and could be very valuable in that respect. Another potential limitation of the algorithm is that it processes each frequency channel independently. While this design choice reduces both the complexity of the algorithm and its computation time, it excludes the possibility for across-frequency processing that could improve performance (Krishnan et al., 2014; Szabó et al., 2016). Finally, animals have been observed to resolve binaural cue ambiguity by having neurons preferentially tune to more reliable spatial cues in different frequency regions (Cazettes et al., 2014). Inspiration could be taken from these observations to improve spatial tuning and overcome spatial leakage. Again, deep-learning based optimization methods may help identify these reliable cues for human listeners and multitalker mixtures.

Future work with the BOSSA model could include both sound segregation and localization by comparing the response of each spatial tuning curve to predict source azimuth, possibly utilizing a denser array of STNs. Another idea we plan to explore in the future is to apply automatic speech recognition systems to optimize the parameters of the algorithm. This optimization can be performed relatively fast before conducting time-consuming psychophysics experiments. During this optimization process we also plan to investigate the effects of varying sound pressure level and source dynamics on BOSSA performance.

## Data availability statement

The raw data supporting the conclusions of this article will be made available by the authors, without undue reservation.

## Ethics statement

The studies involving human participants were reviewed and approved by the Boston University Institutional Review Board. The patients/participants provided their written informed consent to participate in this study.

## Author contributions

KC designed the algorithm under the supervision of KS and HC, conducted the experiment, analyzed the data, and wrote the first version of the manuscript, with editing by AB, VB, HC, and KS. KC and VB designed the psychophysical experiment. AB assisted with additional data analysis. All authors contributed to the article and approved the submitted version.
